# Endogenous circadian temperature rhythms relate to adolescents’ daytime physical activity

**DOI:** 10.3389/fphys.2022.947184

**Published:** 2022-09-07

**Authors:** Liisa Kuula, Jari Lipsanen, Timo Partonen, Jaakko Kauramäki, Risto Halonen, Anu-Katriina Pesonen

**Affiliations:** ^1^ SleepWell Research Program, Faculty of Medicine, University of Helsinki, Helsinki, Finland; ^2^ Department of Psychology and Logopedics, Faculty of Medicine, University of Helsinki, Helsinki, Finland; ^3^ Finnish Institute for Health and Welfare, Helsinki, Finland; ^4^ Cognitive Brain Research Unit, Faculty of Medicine, University of Helsinki, Helsinki, Finland

**Keywords:** sleep, adolescence, actigraphy, accelerometer, thermologger, physical exercise

## Abstract

Circadian rhythms relate to multiple aspects of health and wellbeing, including physical activity patterns. Susceptible circadian regulation predisposes to circadian misalignment, poor sleep, sleep deprivation, increased sleepiness, and thereby sedentary behavior. Adolescents’ circadian regulation is particularly vulnerable, and may lead to sedentary behavior. To investigate which factors associate strongest between physical activity (PA) and circadian behavior, we conducted multimodal circadian rhythm analyses. We investigate how individual characteristics of habitual circadian patterns associate with objectively measured PA. We studied 312 adolescents [70% females) (56% with delayed sleep phase (DSP)], mean age 16.9 years. Circadian period length, temperature mesor (estimated 24 h midline) and amplitude (difference between mesor and peak) were measured using distally attached thermologgers (ibutton 1922L, 3-day-measurement). We additionally utilized algorithm-formed clusters of circadian rhythmicity. Sleep duration, timing, DSP, and PA were measured using actigraphs (GeneActiv Original, 10-day-measurement). We found that continuous circadian period length was not associated with PA, but lower mesor and higher amplitude were consistently associated with higher levels of PA as indicated by mean Metabolic Equivalent (MET_mean_) and moderate-to-vigorous PA (MVPA), even when controlling for sleep duration. Separate circadian clusters formed by an algorithm also reflected distinct patterns of PA accordingly. Late sleepers and those with DSP were less likely to engage in MVPA compared to non-DSP and had more sedentary behavior. Adolescents who engage in higher levels or high-intensity PA have better circadian regulation, as measured by different objective methods including distal temperature measurements as well as actigraphy-measured sleep-wake behavior.

## 1 Introduction

Chronobiology and circadian rhythms have gained accumulating interest in healthcare; specifically their interactions with other processes that govern metabolism and whole-body physiology (Burki, 2017). Exercise biology, including physical activity (PA) intensity, similarly explains mechanisms relating to overall health and metabolism (Adamovich et al., 2021), thus, both PA and circadian rhythms are of interest from a public health perspective.

Both late circadian rhythms as well as sedentary behavior increases mortality and disease risks (De Rezende et al., 2014; Wong et al., 2015; Cliff et al., 2016; Fabbian et al., 2016). The mechanisms underlying these associations are tied to genetics (Dashti et al., 2015; Benna et al., 2017), cardio-vascular functioning (Wong et al., 2015; Nystoriak and Bhatnagar, 2018), metabolism (Depner et al., 2014; Panda, 2016), as well as lifestyle choices (Kanerva et al., 2012; Logan et al., 2018). Interestingly, several mechanisms that pace the body’s circadian rhythm also regulate temperature during physical activity (Waterhouse et al., 2005). While physical activity and sleep behavior are intertwined (Atoui et al., 2021), it remains unclear how individual biological characteristics of habitual circadian patterns are related to objectively measured physical activity.

The central pacemaker of the human body clock is the suprachiasmatic nucleus (SCN). The SCN orchestrates many bodily functions, including body temperature. The body’s core temperature’s circadian rhythm is mainly generated *via* circadian changes in heat loss through the extremities, mediated by vasodilatation of the cutaneous vasculature (Waterhouse et al., 2005). The nocturnal decline of core body temperature (CBT) is strongly related to the release of melatonin (Cagnacci et al., 1992), and distal temperature has a similar but positive linear relationship with melatonin concentration. During sleep, melatonin and distal skin temperatures are high, and CBT is low. While CBT is the most accurately measured with pulmonary artery catheter, esophageal temperature sensors or rectal thermistors, invasive sensors are not feasible in large-scale studies. Distal skin temperature is a known correlate of CBT (Kräuchi and Wirz-Justice, 1994), and measurement devices are easily administered (Hasselberg et al., 2013). However, utilizing distal temperature as a marker of CBT poses some risks in relation to confounding or masking factors, such as ambient temperature, peripheral vasodilation, prior exercise behavior, or body position (Martinez-Nicolas et al., 2013).

Several external cues pace the body’s circadian clocks and light is considered the most important time giver, or zeitgeber (Roenneberg et al., 2007), so spending time outside regularly in bright daylight keeps individuals entrained to the natural light-darkness cycle (Wright et al., 2013; Stothard et al., 2017). Circadian rhythms are partially genetically determined (Schnell et al., 2014; Jones et al., 2019), environmentally cued (Roenneberg and Merrow, 2007), and, to some extent individually controlled (Richardson et al., 2017; Przepiórka et al., 2019). Additionally, aging and normative human development influences these daily patterns: adolescence in particular is a critical time period for the formation of diurnal preferences (Randler et al., 2017; Stolarski et al., 2021), which refer to an individual’s preference for timing their daily activities, as well as behaviors associated with physical activity (Guthold et al., 2020).

In addition to basic research on circadian clocks, studies investigating their associations with PA have emerged recently, especially from the viewpoint of public health. Thus, studying the reciprocal associations between the circadian clock and exercise performance is of growing interest. Research on PA in relation to sleep has mostly focused on the timing, duration, and intensity of PA with the overarching goal to optimize beneficial outcomes for the individual’s health and wellbeing (Kahn et al., 2021; Lang et al., 2022). The timing of PA can alter rest–activity profiles, and affect clock gene expression. This was seen in particular in one study with mice (Adamovich et al., 2021). That study found that daytime variance in capacity for endurance exercise was controlled by circadian clock, especially so that training in the late part of a mouse’s active phase (compared with the early part of the active phase) improved exercise performance, and additionally that training in the later part of the active phase yielded better exercise capacity in mice (Adamovich et al., 2021). This may be implied also in studies on subjective circadian preference and performance levels at certain times of day: those with morning preferences generally show better athletic performances in the morning while evening preference types do so in the evening (Vitale and Weydahl, 2017). In a similar line of findings, a review on the relationship between PA and CBT suggested that since many of the mechanisms that create the circadian rhythm of CBT are the same as those that occur during thermoregulation in exercise, it is likely that optimal performance occurs in specific temperatures; while warm-ups before PA are important for optimal performance, it is noteworthy that increasing already high body temperature may cause fatigue (Waterhouse et al., 2005). Furthermore, studies under strictly controlled conditions have suggested that the variation in temperature’s circadian rhythm is mainly caused *via* heat loss by cutaneous vasculature of the distal limbs rather than metabolic heat production (Kräuchi and Wirz-Justice, 1994). These studies imply a need to consider individual variation in thermoregulation in physical activities, as it seems that optimal performance is related to several factors, and thus an overall “one-size-fits-all” recommendation for PA performance is not feasible.

In studies investigating overall associations between habitual PA and subjective circadian preference, those who have an evening preference typically have lower levels of PA when compared to those with intermediate or morning preferences (Schaal et al., 2010; Urbán et al., 2011; Wennman et al., 2015; Merikanto et al., 2020). Additionally, women who have later or longer sleep rhythms engage in less moderate to vigorous physical activity (MVPA) levels throughout the day (Bailey et al., 2020). These findings suggest a relationship between circadian rhythms and habitual PA patterns, but findings are not fully coherent, likely due to the multiple mechanisms and long time scale which are involved in the development of an individual’s circadian rhythms.

Some studies have (Buxton et al., 1997; Youngstedt et al., 2019; Lang et al., 2022) described the time-dependent exercise-induced phase shift of the onset of melatonin when PA has been timed specifically. This can also be seen in one study done under laboratory conditions: PA in the morning enhanced parasympathetic activity as indicated by heart rate variability, while PA in the evening delayed the circadian rhythm by 1 h (Yamanaka et al., 2015) across a 7-day study protocol. One study stated that evening physical activity alters wrist temperature circadian rhythmicity so that those with PA timed later on in the day had significantly flattened and irregular patterns in circadian temperature (Rubio-Sastre et al., 2014). While these studies highlight the interaction between specifically timed PA and circadian rhythmicity, they also emphasize the need for consideration of individual circadian rhythms in relation to optimal performance.

During the teenage years, biological, social, and psychological pressure tend to delay circadian rhythms, while physical activity levels decrease (Crowley and Eastman, 2018). Delayed Sleep Phase (DSP) has been defined as a subclinical condition with a discrepancy between socially desired wake-up schedules and an individual’s actual sleep-wake patterns, and it commonly remains untreated in healthcare. DSP’s clinical dimension Delayed Sleep Phase Disorder (DSPD) has a prevalence of up to 16% (Murray et al., 2017), and its subclinical forms are likely to be much higher in this age group. Passive lifestyles and decline in physical activity behavior patterns are typically associated with late, poor, and irregular sleep, as has been seen in adults (Kline, 2014; Dubinina et al., 2021), adolescents, and children (Lin et al., 2018; Kim et al., 2020). Correspondingly, higher levels of physical activity usually increase sleep duration and help maintain a regular sleep-wake rhythm, though this association is not necessarily linear (Flausino et al., 2012; Kline, 2014; Pesonen et al., 2022). Despite a number of papers addressing the relationship between exercise and circadian biology, most studies, however, do not consider multiple circadian rhythmicity markers, and thus the characterization of functional interaction between PA and circadian clocks is currently limited.

Given that late circadian rhythms and declining physical activity levels are common features during adolescence, which both negatively influence each other, the objective of the present study is to investigate adolescent circadian rhythms and PA. In addition to late circadian rhythms, sedentary behavior is a common feature of adolescence (Barnett et al., 2018). Our recent study found that those with a circadian preference for eveningness were especially vulnerable for passive lifestyles and increased sedentary behavior during the transition from childhood towards adolescence (Merikanto et al., 2020). Additionally, one of our other studies in adolescents found a strong biological basis for DSP, a continuing tendency to stay awake past desired bedtimes (Lipsanen et al., 2021). Based on these findings, we hypothesize to find associations between biologically determined early circadian rhythms and high levels of PA.

In the current study, our overall objective is to investigate how disruption in adolescent circadian rhythms, as measured by distal, diurnal temperature patterns as well as sleep scheduling, is reflected on objectively measured physical activity. Based on previous studies described above, it is likely that thermoregulation is optimal in those with more PA. We hypothesize to find late circadian rhythms, sleep pattern irregularity, late sleep phase, and both longer and shorter circadian period length to be associated with lower daytime physical activity. Our secondary objective is to investigate how individuals belonging to distinct circadian temperature variation clusters (Lipsanen et al., 2021) differ in their PA profiles. No specific hypothesis is set for these analyses. As a further investigation we also analyze the association of PA and circadian measures while controlling for sleep duration.

## 2 Materials and methods

### 2.1 Participants and procedure

Participants (*n* = 312, 70% female) came from the SleepHelsinki!-cohort which has been described in detail previously (Short et al., 2019; Kuula et al., 2020). The participants were born between 1999 and 2000, and during the time of this data collection were 16.9 years old (SD = 0.6). The original cohort included 1,374 participants (Short et al., 2019; Kuula et al., 2020; Lipsanen et al., 2021), and a subsample was invited to participate in more detailed measurement follow-ups. Based on the online survey questionnaire two subsamples: 1) those without DSP (N = 188) and 2) those with DSP (N = 364) were identified and invited to participate in the measurement phase. The criteria for belonging to the late sleep phase group was reporting bedtime after 1 a.m. at least three times per week. Out of these 552 invited adolescents, 353 agreed to participate and 24 dropped out before completing all the measurements phases. Informed consent was obtained from all participants. All procedures followed were in accordance with the Helsinki Declaration and its later amendments. Ethical permission was obtained from The Hospital District of Helsinki and Uusimaa Ethics Committee for gynaecology and obstetrics, pediatrics and psychiatry (Decision number. 50/13/03/03/2016). The study is registered under Clinical Trials (ID: 1287174). 312 participants provided valid, complete ibutton data (*n* = 281), sleep and PA (actigraphy) data, and questionnaire data from the initial screening.

### 2.2 Morningness eveningness questionnaire

The original questionnaire administered to all 1,374 participants included a short version of the original Morningness Eveningness Questionnaire [MEQ (Horne and Ostberg, 1976)], including six items (4, 7, 9, 15, 17, and 19) which yielded a rMEQ sum score (Basnet et al., 2018). A circadian preference for morningness is indicated by a higher score. The six items had a Cronbach’s alpha of 0.70 in our data. We additionally classified the rMEQ sum score into three categories: Evening circadian preference (5–12 points), Daytime circadian preference (13–18 points), and Morning circadian preference (19–27 points) (Hatonen et al., 2008).

### 2.3 Circadian temperature measurements

We used ibutton 1922L thermologgers to measure circadian oscillations in skin temperature. Thermochron iButtons (DS1922 L, Maxim Integrated, San Jose, CA, United States) are small, light (about 3 g), round, stainless steel data loggers with thermometers. They contain digital thermistor sensors which measure temperature with 0.0625°C resolution and have the accuracy of ±0.5°C from −10°C to +65°C. They include memory for storing data on the temperature and time recordings, and can be initialized to the desired logging frequency. Ibuttons have been validated previously (Hasselberg et al., 2013). In the current study, we selected the measurement rate to be one per minute, and the participants were instructed to wear the device for a minimum of three consecutive days. The iButton was attached onto the inner side of the wrist approximately upon the radial artery using adhesive medical tape. Participants were instructed how to re-attach the iButton if it was removed from the wrist, and advised to write down all times when this was done. The data were read with the USB Port Adapter as connected to the PC 1-Wire Connectivity Reader and extracted with the OneWireViewer software (Maxim Integrated, San Jose, CA, United States).

**FIGURE 1:**
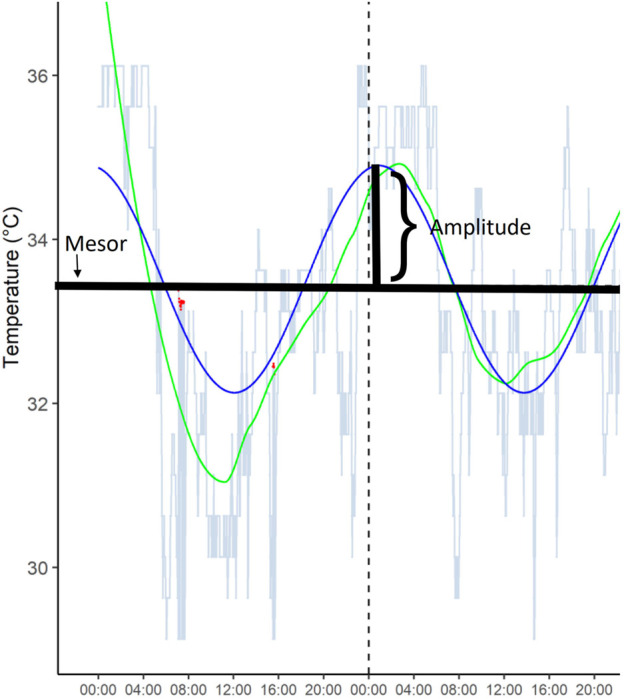
A representative example of temperature amplitude and mesor from one participant over 48 h.

Circadian rhythm measures included amplitude (half the extent of predictable variation within a cycle), mesor (Amplitude Acrophase MESoR, midline estimating statistic of rhythm), and period length (commonly considered to be 24 h). Period length was set to values between 21 and 29 h; other values were considered outliers or measurement errors based on visual inspection of the data. These were derived from 3 days’ and nights’ ibutton measurements. Figure 1 illustrates Mesor and Amplitude derived from temperature measurements.

#### 2.3.1 Cluster analysis from temperature measurements

In the present study, we identified three cluster groups of sleep and circadian profiles based on thermologger data [Cluster 1 (C1) *n* = 100; Cluster 2 (C2) *n* = 130; Cluster 3 (C3) *n* = 51], as reported previously (Lipsanen et al., 2021). These clusters were created with diverse multivariate time-series clustering methods and stochastic learning tools, and they reflect endogenous circadian temperature patterns, as has been reported in detail previously (Lipsanen et al., 2021). As reported previously, all the clusters differed from each other in relation to temperature and amplitude. C1 had the highest average temperature (mean = 34.29) and lowest amplitude (mean = 1.02), while C3 had lowest temperature (mean = 32.80) and, on average, highest amplitude (mean = 2.09). Between these clusters there was C2, whose members had temperatures (mean = 33.46) and amplitudes (mean = 1.38) that fell between those of C1 and C3. Detailed comparisons of the predictive power between the clusters and different Cosinor parameters can be found in Lipsanen et al. (2021). While characteristic qualities overlap across cluster groups, this data reduction technique enables investigating several aspects of circadian regulation within one variable.

### 2.4 Physical activity and sleep actigraphy

The adolescents’ sleep and PA were measured using actigraphy (GENEActiv Original, Activinsights Ltd., Kimbolton, United Kingdom) for 10 days and nights (M = 8.0, SD = 1.6). GENEActiv Original actigraphs are wrist-worn, tri-axial accelerometers which can be initialized to collect raw 12-bit MEMS acceleration data at selected frequencies. Participants were instructed to wear the actigraph device on their non-dominant wrist for 7–10 consecutive days and nights. They were given a sleep log booklet to fill in alongside the actigraphy measurement period, and were instructed to write down sleep onset and offset times, as well as all times when the device was not worn on the wrist. The devices were set to sample activity at a frequency of 50 Hz, and their data were downloaded onto a computer and aggregated into 30-s epochs.

#### 2.4.1 Physical activity and sedentary behavior

Physical activity was operationalized as mean metabolic equivalents (MET_mean_), which was calculated from GeneActiv raw accelerometer data, which consists of time-stamped *x*, *y*, and *z* axes acceleration data in 12-bit resolution, in gravitational units (g) including directionality (negative or positive sign, raw range of −2048 to +2047). As our participants were close to adult age, we formed intensity categories based on validated adult cut-points (Esliger et al., 2011), and then classified activity into three categories: sedentary, light PA, and MVPA. We calculated how many hours the participant had spent on average in that intensity category.

#### 2.4.2 Sleep

The sleep data handling procedure has been described previously (Kuula et al., 2020). The sleep variables utilized in this study included sleep duration, sleep midpoint, sleep duration irregularity (SD), and sleep midpoint irregularity (SD). These were derived as mean measures over the entire logging period (M = 8 nights, SD = 1.6) in order to detect typical sleep. Participants were instructed to follow their normal sleeping patterns over the measurement period, and to report any atypical events or illnesses. The sleep actigraphy measurement included both weekdays as well as weekends with no differentiation between the two. Finnish school mornings may start as early as 8:00 a.m. or as late as 10:20 a.m., and it is common that weekend morning have schedules, too. Our data did not include information on morning schedules, so all nights and morning are treated as equal regardless of weekday.

The actigraphy data were cleaned from artefacts as described previously (Kuula et al., 2020). Only those with three or more nights of data were included. Sleep duration was calculated as the assumed total sleep time (i.e., the amount of time between sleep onset and wake-up time). Sleep timing was assessed by calculating the mean midpoint of the entire sleep period between sleep onset and offset. As measures of sleep regularity, we calculated the individual standard deviation of sleep duration and sleep midpoint over the measurement period. Finally, as a measure of DSP, we formed a binary variable according to weekly bedtimes: those who went to bed at 1 a.m. or later on 3 or more nights per week were considered to have DSP. DSP is a subclinical condition with a discrepancy between desired wake-up schedules and actualised rhythms, and while there are no official reference values for DSP, we set the limit of 1 a.m. based on a previous study in a similar age group (Danielsson et al., 2016).

### 2.5 Statistical methods

Initial comparisons between sexes were done using Chi squared test for categorical variables and *t* tests for continuous variables. We used multiple regression analyses to investigate associations between circadian markers derived from temperature measurements and different intensity PA levels. Additionally, we divided the temperature patterns by dividing them into thirds, and then compared differences in PA across the tertiles formed this way. Furthermore, we compared the PA levels of rMEQ preference groups, different cluster members as well as those adolescents with DSP to those with no DSP by using two-way ANOVAs. The associations between all continuous sleep and physical activity measurements were investigated using regression analyses. As a further investigation we also analysed the association of PA and circadian measures while controlling for sleep duration. Alpha was set at 0.05 and all analyses were done with IBM Statistics SPSS 26.

## 3 Results

### 3.1 Initial comparisons

The sample’s characteristics and comparisons between female and male sexes are presented in Table 1.

**TABLE 1:** Descriptive characteristics of the sample.

Characteristic	N (%)/Mean (SD)	Female N (%)/Mean (SD)	Male N (%)/Mean (SD)	F/χ^2^	*p*
Age	312	219 (70)	93 (30)		
	16.9 (0.6)	16.8 (0.6)	16.9 (0.6)	2.82	0.328
Delayed Sleep Phase (yes)	172 (56)	108 (50)	64 (69)	9.60	0.002
rMEQ score	13.0 (3.9)	12.7 (4.0)	13.5 (3.5)	1.61	0.116
Evening circadian preference	121 (48)	110 (51)	38 (42)	1.70	0.427
Daytime circadian preference	115 (46)	91 (42)	45 (50)		
Morning circadian preference	17 (7)	15 (7)	7 (8)		
Actigraphy measurements					
Sleep duration (hh:min)	7:43 (0:58)	7:50 (0:56)	7:28 (1:02)	0.18	0.003
Sleep midpoint (hh:min)	4:46 (1:14)	4:38 (1:13)	5:07 (1:14)	0.05	0.002
Irregular sleep duration (hh:min)	1:28 (0:38)	1:28 (0:38)	1:29 (0:39)	0.01	0.812
Irregular sleep timing (h)	1.23 (0.55)	1.20 (0.54)	1.31 (0.57)	0.22	0.121
Thermologger measurements					
Circadian period length (h)	25.36 (1.15)	25.39 (1.10)	25.29 (1.26)	2.17	0.500
Mesor (°C)	33.63 (0.61)	33.64 (0.64)	33.60 (0.54)	4.94	0.596
Amplitude	1.38 (0.54)	1.48 (0.56)	1.13 (0.42)	5.77	<0.0001
Mean Physical Activity					
MET	1.28 (0.19)	1.29 (0.18)	1.27 (0.21)	3.76	0.496
Sedentary	10.18 (1.61)	10.07 (1.66)	10.42 (1.46)	1.25	0.075
Light	0.96 (0.31)	0.99 (0.28)	0.90 (0.35)	2.21	0.020
Moderate to vigorous (MVPA)	1.90 (0.72)	1.92 (0.69)	1.84 (0.78)	1.65	0.366

Abbreviations: rMEQ, Reduced Morningness Eveningness Questionnaire sum score; MET, metabolic equivalents. P refers to difference between females and males; F statistic reported for continuous variables; χ2 for categorical variables.

### 3.2 Associations between physical activity and temperature measurements

Table 2 presents the associations between physical activity levels and circadian period length, mesor, and amplitude. These analyses show that lower mesor and higher amplitude are associated with MET_mean_, light PA, and MVPA (all *p*-values <0.001).

**TABLE 2:** Multiple regression analyses showing associations between circadian markers derived from temperature measurements and physical activity levels.

*PA level*		B	Std. Error	*p*	Lower bound	Upper bound
MET_mean_	Period	0.00	0.01	0.72	−0.02	0.02
	Mesor	−0.05	0.02	<0.001	−0.09	−0.02
	Amp.	0.07	0.02	<0.001	0.03	0.11
Sedentary	Period	−0.07	0.09	0.45	−0.24	0.11
	Mesor	−0.15	0.17	0.38	−0.47	0.18
	Amp.	−0.30	0.19	0.11	−0.67	0.06
Light	Period	0.00	0.02	0.80	−0.04	0.03
	Mesor	−0.10	0.03	<0.001	−0.16	−0.05
	Amp.	0.13	0.03	<0.001	0.07	0.19
MVPA	Period	−0.02	0.04	0.67	−0.09	0.06
	Mesor	−0.23	0.07	<0.001	−0.37	−0.08
	Amp.	0.38	0.08	<0.001	0.23	0.54

Abbreviations: Amp., Amplitude; PA, Physical Activity; MVPA, Moderate to Vigorous Physical Activity; MET, metabolic equivalents; B, Coefficient.

Table 3 presents associations between sleep measures and physical activity levels. When controlling for sleep duration as a confounder between PA and circadian markers derived from temperature measurements, we found that adding sleep duration into the model attenuated the association between MET_mean_ and amplitude to non-significant (*p* = 0.075). The complete results for this additional analysis are presented in Supplement Table S1.

**TABLE 3:** Multiple regression analyses showing associations between sleep measures and physical activity levels.

	MET_mean_	Sedentary	Light PA	MVPA
*Sleep variables*	**B (95% CI)**	**B (95% CI)**	**B (95% CI)**	**B (95% CI)**
Duration	−0.01 (−0.03, 0.01)	**−0.59 (−0.76, −0.42)**	−0.02 (−0.06, 0.01)	−0.01 (−0.09, 0.07)
Midpoint	**−0.03 (−0.05, −0.02)**	**0.22 (0.07, 0.36)**	**−0.05 (−0.08, −0.02)**	**−0.15 (−0.22, −0.09)**
Irregular duration	−0.01 (−0.04, 0.02)	0.03 (−0.25, 0.31)	−0.04 (−0.09, 0.02)	−0.08 (−0.21, 0.04)
Irregular timing	−0.03 (−0.06, 0.01)	**0.52 (0.20, 0.84)**	−0.05 (−0.11, 0.01)	**−0.17 (−0.31, −0.03)**

Abbreviations: PA, Physical Activity; MVPA, Moderate to Vigorous Physical Activity; MET, metabolic equivalents; B, Coefficient; 95 % CI, 95 % Confidence Interval. Significant associations (*p* < 0.05) are bolded.

**FIGURE 2:**
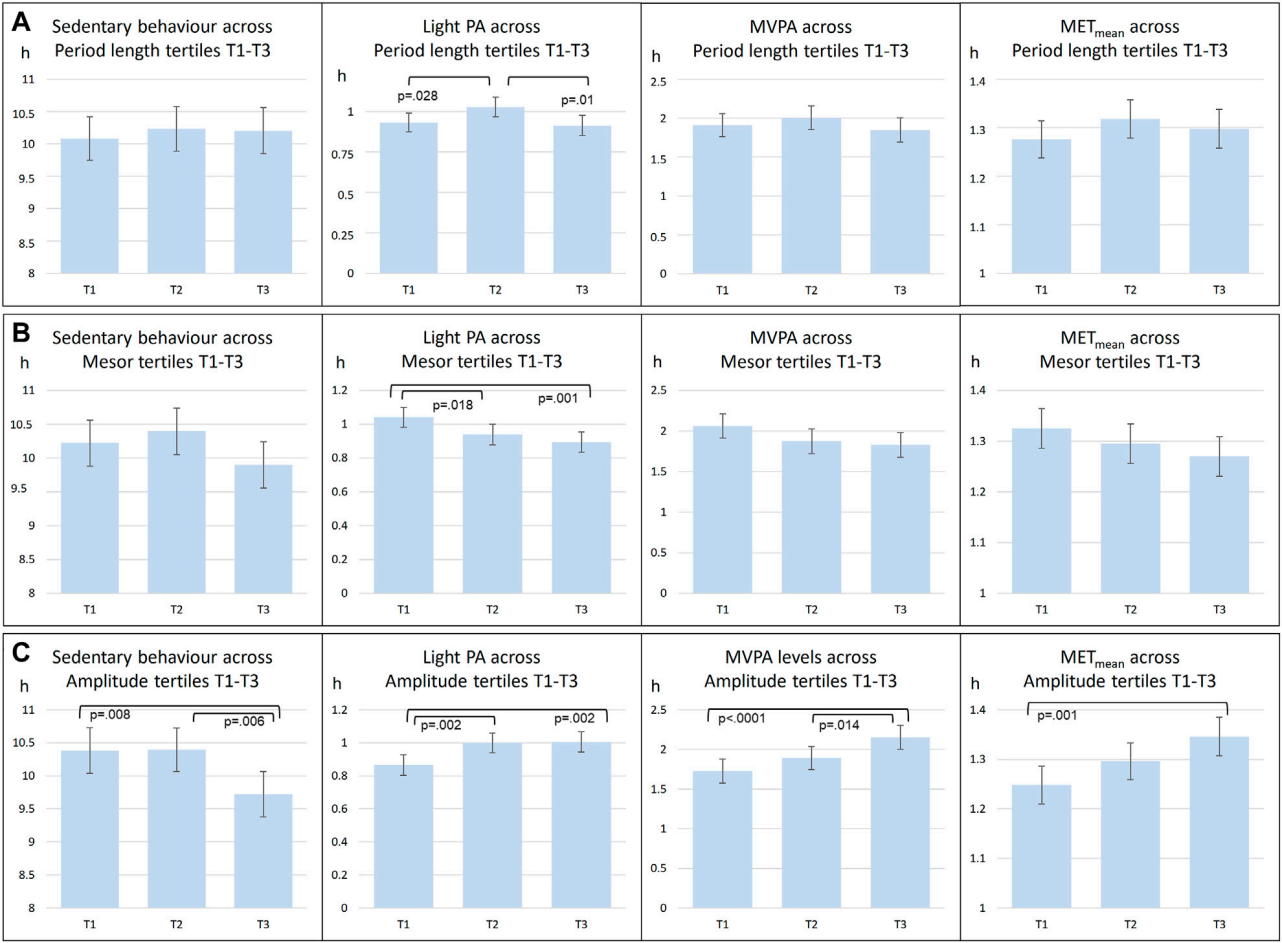
Comparisons of mean level PA across circadian period length **(A)**, Mesor **(B)**, Amplitude **(C)** tertiles (T1-T3). abrAbbreviations: PA, Physical Activity; MVPA, Moderate to Vigorous Physical Activity; MET, metabolic equivalents.

**FIGURE 3:**
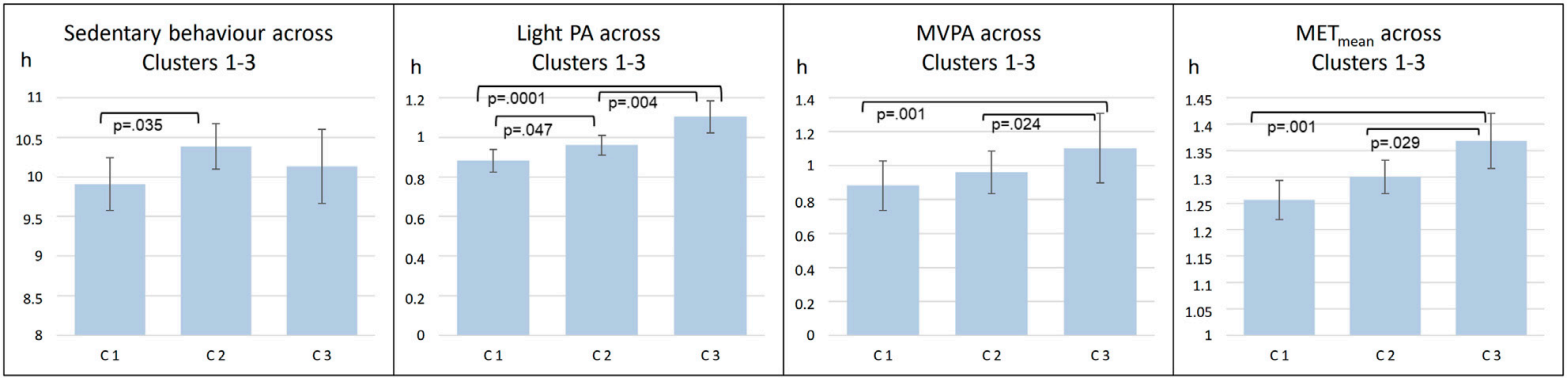
Comparisons of mean level PA across three clusters (C1-C3). abrAbbreviations: C1 Cluster 1; C2, Cluster 2; C3, Cluster 3; PA, Physical Activity; MVPA, Moderate to Vigorous Physical Activity; MET, metabolic equivalents.

In further analyses, circadian temperature measures were divided into tertiles, which were then compared in relation to PA intensity (Figure 2). Tertiles were divided as follows: Amplitude T1 < 1.097; T2 1.097–1.549; T3 > 1.549; Period T1 < 24.97; T2 24.97–25.83; T3 > 25.83; Mesor T1 < 33.35; T2 33.35–33.88; T3 >33.88. These analyses point to the same direction as continuous measures of circadian rhythms: those with less sedentary behavior and more PA had higher amplitude and lower mesor. Furthermore, clusters were similarly compared (Figure 3). Clusters compared against each other showed similar directions, with those belonging to C3 having significantly more MVPA than those in other clusters, and those belonging to C2 having most sedentary behavior.

### 3.3 Associations between delayed sleep phase and physical activity

There were no differences in MET_mean_ between DSP and non-DSP groups (*p* = 0.41). When comparing PA in different intensity groups, those adolescents with DSP symptoms had less MVPA compared to non-DSPs [1.8 vs. 2.0, *t* (304) = 2.337, *p* = 0.02] and more sedentary behavior [10.6 vs. 9.8, *t* (304) = -4.696, *p* < 0.0001]. Light PA levels did not differ between those with DSP characteristics and those without (*p* = 0.16).

### 3.4 Associations between the Morningness-Eveningness sum score, circadian preference, and physical activity

A higher continuous rMEQ sum score (indicating preference for morningness) was associated with a greater amount of light PA levels [*R* ^2^ = 0.024, F (1, 304) = 7.59, *p* = 0.006], but not regarding MET_mean_ (*p* = 0.137), sedentary behavior (*p* = 0.088), or MVPA (*p* = 0.071). There were no differences between categorized rMEQ scores of circadian preference and PA (all *p*-values >0.28).

## 4 Discussion

We studied associations between objective measurements of PA and circadian patterns by utilizing distal circadian temperature data, actigraphy sleep data, and self-reported circadian preference, and found that the distal circadian temperature rhythm was strongly associated with PA whereas sleep duration and subjective reports of circadian preference were not. In line with our hypotheses, distal circadian temperature measurements indicated that higher amplitude of circadian temperature variation and lower mesor (i.e., lower level of overall temperature) were both significantly associated with higher levels of PA as shown by higher levels of MET_mean_, MVPA, and light PA.

From the individual circadian characteristics, the period length was not associated with the intensity of PA. However, we previously identified three very distinct clusters of individuals based on their circadian temperature dynamics. This approach enabled characterisation of the entire dynamics of the circadian time series data. Cluster 3 was characterized by the greatest amplitude of circadian temperature, earliest acrophase and lowest mesor, and basing on the concurrent validity indicators, it associated with the most regular and earliest-timed sleep–wake pattern. In the present analyses, we found that Cluster 3 members had the most active lifestyles as measured by all PA intensity levels and METmean. The two other clusters (Cluster 1 and Cluster 2) were lower in amplitude, higher in mesor, and had later sleep midpoints and more irregular sleep timing when analyzing their actual sleep–wake rhythms (Lipsanen et al., 2021) In the current study, Cluster 2 members had the most sedentary behavior.

The majority of these associations between circadian temperature measurements and PA remained significant controlling sleep duration, suggesting that the circadian component is a strong associate with exercise and PA regardless of the amount of habitual sleep. Circadian patterns in temperature oscillations are hypothalamically controlled, and form an important rhythm for several functions relating to whole-body physiology and metabolism. While our study is correlational in nature, PA influences thermoregulation greatly, and thus our findings may indicate real-life associations between habitual PA and circadian regulation.

Circadian temperature measurements indicated that higher amplitude and lower mesor were significantly associated with higher levels of PA, as shown by higher levels of MET_mean_, MVPA, and light PA. These findings are well in line with our hypothesis regarding circadian regulation and the PA intensity. Furthermore, these results support the notion of PA as thermoregulatory enhancer of body clocks. Regarding cluster analyses, we found that Cluster 3 members seemed to have the most active lifestyles as measured by all PA intensity levels and MET_mean_. Cluster 2 members had the most sedentary behavior. The cluster findings are likely to be explained by the same individual circadian temperature variables (mesor, amplitude, period length) which provided evidence already on their own, but they further emphasize the concept of a phenotype which may be associated with successful thermoregulation and optimal circadian patterns.

Our findings were mostly in the realm of circadian markers rather than sleep duration. Sleep duration may include more noise stemming from fluctuating sleep need, while sleep timing and circadian rhythms indeed have a solid biological basis, which would be seen more readily in temperature measurements. The current study did not find differences in PA between categorized subjective circadian preference types, contrary to previous studies (Schaal et al., 2010; Urbán et al., 2011; Merikanto et al., 2020). While we did not find significant differences between all PA levels, the directions point to similar directions as previous studies, and indeed those with a preference for morningness had more light PA levels. While a preference for morningness may protect from harmful lifestyle choices, it seems that DSP plays a bigger part in the current study: those categorized as having DSP characteristics in our study had less MVPA and more sedentary behavior.

Similar to melatonin, CBT can be considered an internal pacemaker, which synchronizes the body’s different circadian oscillators and functions. CBT is known to synchronize peripheral circadian rhythms in mammals (Buhr et al., 2010), and it is regulated by a complex feedback system with only a few Celsius (°C) degrees’ variation. CBT has optimal values, which are kept by the hypothalamic thermoregulatory centre. Optimal thermoregulation is achieved by activating relevant mechanisms relating to two hypothalamic groups of neurons, which induce either heat loss or heat gain (Zhao et al., 2017). A recent study reported finding subsets of temperature-activated neurons in the preoptic area nuclei and dorsomedial hypothalamus nuclei, and showed that modulating their activity can lead to alterations in core temperature (Zhao et al., 2017). Furthermore, the study suggested that heat-activated GABAergic neurons in the preoptic area nuclei may reduce the activity of cold-activated neurons in the dorsomedial hypothalamus nuclei, which function to increase both thermogenesis as well as PA. While there are several bodily mechanisms of heat production (e.g., metabolic, PA, or muscle non-shivering thermogenesis), and while ambient temperature or high intensity PA provide a wide range of challenges, the hypothalamic thermoregulation centre usually manages to maintain CBT at a rather stable level. Thus, neural thermoregulation is a key function in whole-body physiology and metabolism, and it is likely that through this function, PA supports body clocks (Buhr et al., 2010).

From a public health point of view, it is important to consider how individuals could be supported in finding their optimal PA levels as well as sleep-wake rhythms. Adolescence may be a particularly turbulent developmental period regarding circadian rhythms as well as PA. When put together, these may lead to late sleep rhythms which can deprive teenagers from natural sources of bright light. Further research is needed to study how thermoregulation supports optimal physical performance in relation to sleep-wake rhythms. While it may well be that those with an active lifestyle are also able to maintain regular sleep-wake rhythms, an equally feasible explanation is that healthy sleep enables desirable levels of PA, or, that optimal PA and circadian regulation belong to the same phenotype of a balanced capability of thermoregulation, or perhaps even psychological self-regulation. Longitudinal analyses will aid in detecting the temporal pathway behind our findings.

While thermologgers such as the ibuttons used in this study are accurate devices per se (Hasselberg et al., 2013), their measurements may reflect other external influences, such as ambient temperature or peripheral vasodilatation. Furthermore, the intensity of physical activity is likely to increase temperature. Therefore, our findings should be interpreted with caution, as they are correlational in nature and may be influenced by environmental factors as well as everyday behavioral choices. As one additional limitation, our study did not include measurements of time spent in bright light or outside. As light is one of the most important cues for circadian clock oscillations, it is likely to explain some of our findings. However, light exposure alone is not a sufficient explanatory mechanism for the relationship between PA and circadian rhythms, and it is known that activity-rest patterns may influence circadian timing independently of light (Richardson et al., 2017).

The functional interaction between physical activity and circadian clocks in everyday life is only partially characterized. The complex processes between sleep behavior and circadian rhythms are influenced by genetic, environmental and habitual factors, and thermoregulation is likely to play a part in these mechanisms.

### 4.1 Strengths and limitations

A strength of this study was including several objective measurements of circadian patterns, which enable comparing endogenous rhythms and actualized patterns of rest and activity. We were also able to compare PA across subjective circadian preferences which are known risks for several pathologies and diseases.

As a limitation we did not consider meals or information on eating behavior, or, other relevant external time-givers. Our study did not include genetic information of the participants, which would have enabled comparisons of different mechanisms of circadian rhythm throughout development, and whether these influence PA intensity in differentiated ways. As a minor limitation, we were unable to reliably distinguish between free days and work/school days. One limitation relating to our devices is the masking effect which is likely to explain some of our findings. As the mechanisms relating to CBT’s circadian rhythm are similar to those of thermoregulation during PA, it is likely that the interaction between these two phenomena explains some of our results (Waterhouse et al., 2005). This poses a risk in interpreting our results, as the rhythm of core temperature is also affected by the sleep-wake cycle. In future studies this and other masking effects could be overcome by utilizing constant routines, which deprives the participant from sleep and variation in timed activities and behaviors. However, the current study aimed to investigate adolescents’ circadian rhythms’ relationship with PA using a natural study setting, and thus introducing constant routines was not an option.

## 5 Conclusion

Based on the current study, we conclude that several aspects of late circadian rhythms and actualized late sleeping schedules are associated with lower levels of physical activity. Circadian temperature measurements as well as actigraphy-measured sleep-wake behavior reflect habitual PA levels. Adolescents face challenges in maintaining optimal circadian rhythmicity, but sufficient PA is likely to support this.

## Data Availability

The raw data supporting the conclusions of this article will be made available by the authors upon reasonable request.
