# Supplementary material for: Endogenous circadian temperature rhythms relate to adolescents’ daytime physical activity
Source: Front Physiol. 2022 Sep 7;13:947184. doi: 10.3389/fphys.2022.947184 (PMC9490324; doi:10.3389/fphys.2022.947184)
Supplement: Supplementary file 1 [file DataSheet1.PDF]

Table S1. Multiple regression analyses showing associations between circadian markers derived from temperature measurements and physical activity levels after controlling for sleep duration.

PA level		**B**	**Std. Error**	**p**	**Lower Bound**	**Upper Bound**
MET_{mean}	Period	-0.06	0.08	0.496	-0.22	0.11
	Mesor	-0.34	0.16	0.038	-0.65	-0.02
	Amp.	0.32	0.18	0.075	-0.03	0.68
Sedentary	Period	-0.03	0.10	0.808	-0.23	0.18
	Mesor	0.26	0.20	0.195	-0.14	0.66
	Amp.	-0.36	0.23	0.114	-0.80	0.09
Light	Period	0.00	0.02	0.950	-0.03	0.03
	Mesor	-0.08	0.03	0.016	-0.14	-0.02
	Amp.	0.11	0.04	0.003	0.04	0.18
MVPA	Period	-0.06	0.06	0.339	-0.18	0.06
	Mesor	-0.38	0.12	0.001	-0.61	-0.15
	Amp	0.49	0.13	<0.001	0.23	0.75

Abbreviations: Amp.=Amplitude; PA=Physical Activity; MVPA=Moderate to Vigorous Physical Activity; MET= metabolic equivalents; B=Coefficient.
